# Microbiome composition indicate dysbiosis and lower richness in tumor breast tissues compared to healthy adjacent paired tissue, within the same women

**DOI:** 10.1186/s12885-021-09074-y

**Published:** 2022-01-03

**Authors:** Maria Valeria Esposito, Bruno Fosso, Marcella Nunziato, Giorgio Casaburi, Valeria D’Argenio, Alessandra Calabrese, Massimiliano D’Aiuto, Gerardo Botti, Graziano Pesole, Francesco Salvatore

**Affiliations:** 1 Department of Molecular Medicine and Medical Biotechnologies, University Federico II, Via Sergio Pansini, 5, 80131 Napoli, NA Italy; 2 CEINGE - Biotecnologie Avanzate, Via Gaetano Salvatore, 486, 80145 Napoli, Italy; 3 Institute of Biomembranes, Bioenergetics and Molecular Biotechnologies, Consiglio Nazionale delle Ricerche, Via Giovanni Amendola, 122/O, 70126 Bari, BA Italy; 4 Evolve Biosystems, Inc, 95618 Davis, CA USA; 5 Department of Human Sciences and Quality of Life Promotion, San Raffaele Open University, Via di Val Cannuta, 247, 00166 Rome, Italy; 6 Department of Senology, Istituto Nazionale Tumori - IRCCS, ’Fondazione Pascale’, Via Mariano Semmola, 53, 80131 Napoli, NA Italy; 7 Clinica Villa Fiorita, Via Filippo Saporito, 24, 81031 Aversa, CE Italy; 8 Scientific Directorate, Istituto Nazionale Tumori, Fondazione G. Pascale, IRCCS, Via Mariano Semmola, 53, 80131 Napoli, NA Italy; 9 Department of Biosciences, Biotechnology and Biopharmaceutics, University of Bari “A. Moro”, Piazza Umberto I, 1, BA 70121 Bari, Italy

**Keywords:** Breast cancer microbiome, Microbial dysbiosis, Breast cancer tissues, Next generation sequencing, Breast healthy tissues, Microbiome composition, cancer/healthy paired samples, 16S rRNA

## Abstract

**Background:**

Breast cancer (BC) is the most common malignancy in women, in whom it reaches 20% of the total neoplasia incidence. Most BCs are considered sporadic and a number of factors, including familiarity, age, hormonal cycles and diet, have been reported to be BC risk factors. Also the gut microbiota plays a role in breast cancer development. In fact, its imbalance has been associated to various human diseases including cancer although a consequential cause-effect phenomenon has never been proven.

**Methods:**

The aim of this work was to characterize the breast tissue microbiome in 34 women affected by BC using an NGS-based method, and analyzing the tumoral and the adjacent non-tumoral tissue of each patient.

**Results:**

The healthy and tumor tissues differed in bacterial composition and richness: the number of Amplicon Sequence Variants (ASVs) was higher in healthy tissues than in tumor tissues (p = 0.001). Moreover, our analyses, able to investigate from phylum down to species taxa for each sample, revealed major differences in the two richest phyla, namely, Proteobacteria and Actinobacteria. Notably, the levels of Actinobacteria and Proteobacteria were, respectively, higher and lower in healthy with respect to tumor tissues.

**Conclusions:**

Our study provides information about the breast tissue microbial composition, as compared with very closely adjacent healthy tissue (paired samples within the same woman); the differences found are such to have possible diagnostic and therapeutic implications; further studies are necessary to clarify if the differences found in the breast tissue microbiome are simply an association or a concausative pathogenetic effect in BC. A comparison of different results on similar studies seems not to assess a universal microbiome signature, but single ones depending on the environmental cohorts’ locations.

**Supplementary Information:**

The online version contains supplementary material available at 10.1186/s12885-021-09074-y.

## Background

Breast cancer (BC) is the most common form of cancer among women and, after ovarian cancer, is the second cause of death due to a neoplastic disease worldwide [1, 2]. Familial forms of BC represent up to 20% of all BCs: among these more than 25% are due to predisposing mutations in the *BRCA1/2* genes [3–9] while another percentage concerns mutations in high, moderate and low susceptibility genes [10]. Despite this genetic component, the etiology of up to 80-85% of tumors remains unknown and thus they are considered sporadic. In this context, environmental and lifestyle factors might also modify cancer risk in both familial and sporadic BCs. Nevertheless, most of the factors contributing to BC are still not completely understood thereby limiting BC prevention and treatment measures [11, 12].

The human microbiome plays an important role in promoting health and preventing disease, which suggests that microbial dysbiosis could contribute to increasing the risk of cancer [13–19]. In this regard, in recent years attention has focused on the relationship between the human microbiome and carcinogenesis to assess its role in BC onset and/or development [20–24]. Therefore, in this scenario, we analyzed (in paired samples from the same subject) the microbiome of tumor breast tissue and the adjacent normal one of women affected by BC in the attempt to get a closer view which may shed light on the potential involvement of microbial dysbiosis in breast cancer. To this aim, we used next-generation-sequencing (NGS)-based methodology to analyze the 16 s ribosomal RNA of the microbiome tissue populations.

## Methods

### Patients’ samples and ethics

Biological samples and clinical data were obtained from a total of 34 women attending the Breast Unit of the “Istituto Nazionale dei Tumori - Fondazione G. Pascale” of Naples starting in 2014 lasting 5 years (Table 1). All patients gave their written informed consent to the study that was carried out according to the tenets of the Helsinki Declaration and approved by the Istituto Nazionale Tumori - Fondazione G. Pascale Ethics Committee (protocol number 3 of 03/25/2009). All patients were previously screened for BRCA1/2 mutations using the protocol and the selection criteria reported by D’Argenio et al. 2015 [25].

**Table 1:** Anamnestic and clinical features of patients selected for this study

ID	Under/Over 40 at the onset	Disease Status	BC Familiarity	BRCA1/2 Mutational status *	Tissue Histology	Menarche Age	Pregnancies	Other Features
P1	OVER	breast cancer	no	Wt	nr	nr	nr	nr
P2	UNDER	breast cancer	yes	Wt	luminal A	12	1	oral contraceptives
P3	OVER	LABC	yes	Wt	Her2 related	13	0	oral contraceptives
P4	OVER	LABC	no	Wt	TNBC	11	3	smoke
P5	UNDER	breast cancer	yes	Wt	luminal A	11	2	nr
P6	OVER	breast cancer	yes	Wt	luminal A	11	1	smoke, obesity, ovarian stimulation
P7	OVER	breast cancer	yes	Wt	nr	11	3	smoke, oral contraceptives
P8	UNDER	LABC	yes	Wt	TNBC	14	3	smoke
P9	UNDER	breast cancer	Yes	BRCA1	TNBC	12	3	nr
P10	UNDER	breast cancer	yes	Wt	luminal A	9	0	oral contraceptives
P11	UNDER	breast cancer	no	Wt	nr	11	2	oral contraceptives
P12	UNDER	breast cancer	yes	Wt	luminal A	9	1	nr
P13	OVER	breast cancer	yes	Wt	nr	11	1	ovarian stimulation
P14	OVER	LABC	yes	Wt	nr	14	3	nr
P15	UNDER	breast cancer	Yes	BRCA2	luminal A	16	1	oral contraceptives, smoke
P16	UNDER	breast cancer	yes	Wt	luminal A	10	3	nr
P17	UNDER	breast cancer	no	Wt	luminal A	12	2	oral contraceptives, smoke
P18	UNDER	breast cancer	no	Wt	Her2 related	11	0	smoke
P19	OVER	LABC	yes	Wt	luminal A	14	nr	nr
P20	UNDER	breast cancer	no	Wt	luminal B	12	1	oral contraceptives
P21	UNDER	breast cancer	yes	Wt	luminal B	13	0	oral contraceptives, smoke
P22	OVER	LABC	yes	Wt	nr	12	2	smoke
P23	UNDER	breast cancer	yes	Wt	nr	13	2	nr
P24	OVER	breast cancer	yes	Wt	nr	11	2 (1 abort.)	nr
P25	UNDER	breast cancer	no	Wt	luminal B	16	1	oral contraceptives, smoke
P26	OVER	LABC	yes	Wt	luminal A	14	2	nr
P27	UNDER	breast cancer	Yes	BRCA2	luminal B	13	2	oral contraceptives
P28	UNDER	breast cancer	yes	Wt	nr	13	0	nr
P29	UNDER	breast cancer	no	Wt	luminal B	13	nr	nr
P30	UNDER	breast cancer	yes	Wt	Her2 related	12	1	oral contraceptives
P31	UNDER	breast cancer	yes	Wt	nr	12	2	oral contraceptives
P32	UNDER	breast cancer	no	Wt	nr	12	6	smoke
P33	UNDER	breast cancer	yes	Wt	luminal B	14	2	ovarian stimulation
P34	UNDER	breast cancer	no	Wt	luminal B	13	1	nr

LABC is locally advanced breast cancer (n = 7 patients); TNBC is triple negative breast cancer; BRCA mutated patients (n = 3); nr: not reported

Tumor tissues and healthy tissues, singly paired from the same woman, and surgically removed at the same time (within the same sequencing run, see below), were analyzed for a total of 68 samples, from which total DNA was isolated. Only fresh frozen tissues were used. The tissues were frozen immediately after removal directly in the surgery room to avoid environmental contamination.

To precisely ensure the histology of tissues, all were analyzed in the pathology laboratory (see Table 1).

### Genomic DNA extraction from breast tissue

DNA was extracted from tissues using the QIAamp DNeasy Blood & Tissue kits (Qiagen, Hilden, Germany), according to the manufacturer’s instructions. DNA was quantified using the NanoDrop 2000c Spectrophotometer (Thermo Fisher Scientific, Waltham, MA, USA) and the Qubit dsDNA BR and HS assay kit (Life Technologies, CA, USA).

### Preparation of the 16 S Metagenomic Sequencing Library

Amplification of the V4-V6 regions of the 16 S rRNA bacterial genes was assessed in two PCR steps: a template of 5 ng/µl of DNA for each sample was used for the first PCR, which was performed using the V4-V6 region specific primers with overhang adapters attached. The primer sequences used in this study are listed in Table 2; the primers were designed and synthetized in our core facility.

**Table 2:** Primers used to amplify the V4-V6 regions encoding for the 16 S rRNA for sequencing library preparations

ID of the 16 S primer	Sequence
Forward 16 S V4-V6	TCGTCGGCAGCGTCAGATGTGTATAAGAGACAG**CAGCAGCCGCGGTAATAC**
Reverse 16 S V4-V6	GTCTCGTGGGCTCGGAGATGTGTATAAGAGACAG**TGACGACAGCCATGC**

Illumina 16 S PCR primers with overhang adapters and sequences complementary to V4-V6 regions (in bold)

Subsequently, 1 µl of the PCR product was analyzed on a Bioanalyzer DNA 1000 chip (Agilent, Santa Clara, CA, USA) to verify its size (~550 bp). Next, Agencourt AMPure XP beads (Beckman Coulter, Brea, CA, USA) were used to purify the 16 S V4-V6 amplicons away from free primers and primer dimer species. Purification products underwent further quality and quantity controls by Bioanalyzer DNA 1000 analysis (Agilent, Santa Clara, CA, USA). The second PCR, performed as per the Nextera XT protocol (Illumina, San Diego, CA), allowed the addition of the Illumina sequencing adapters and the dual-index primers, which barcoded each sample. The V4-V6 amplified regions of each patient were purified through Agencourt AMPure XP Beads (Beckman Coulter, Brea, CA, USA), quantified using the Qubit HS assay kit (Life Technologies, CA, USA) and quality-assessed using a High Sensitivity Chip on the 2100 Bioanalyzer Instruments (Agilent, Santa Clara, CA, USA). However, up to 68 libraries were pooled together for sequencing. Therefore, 8 pM of denatured libraries were combined to 25% of 8 pM PhiX control and loaded into the MiSeq v3 reagent cartridge. Sequencing reactions were per-formed through the Illumina MiSeq System (PE 300 × 2), by obtaining an average read length of about 300 bp. The raw sequencing data are available in the SRA repository under the BioProject PRJNA759366.

### Bioinformatic Analysis and Statistics

The Illumina MiSeq paired-end (PE) reads were denoised using a procedure relying on the inference of the Amplicon Sequence Variants (ASVs) (i.e. an estimation of the actual amplicons). The PE reads were treated with cut adapt to remove Illumina adaptors [26]. The trimmed reads were merged using PEAR [27]. The resulting merged reads were denoised by applying the DADA2 workflow [28]. This procedure included the chimera- (i.e. PCR artifacts) and PhiX- (i.e. the PhiX phage is used during Illumina library preparation to increase nucleotide variability) removal [29–31]. ASV sequences were mapped against the human genome (release hg19) using bowtie2 to remove nonspecific amplification products (i.e. 16 S rRNA mitochondrial gene) [32].

The ASVs obtained were taxonomically annotated in BioMaS using the Ribosomal (RDP) database (release 11.5) and the NCBI taxonomy as 16 S rRNA reference collection and taxonomy, respectively [33–36]. In particular, the query sequences were aligned to the reference collection using bowtie2, and the resulting alignments were filtered according to query coverage (≥ 70%) and identity percentage (≥ 90%). A phylogenetic tree was inferred using the QIIME2 align-to-tree-mafft-fasttree plugin: a multiple sequence alignment of ASV sequences was obtained by using MAFFT and the phylogenetic tree was inferred by applying the maximum-likelihood procedure implemented in Fasttree 2 [37–39].

The taxonomic classification was performed using TANGO [40]. In particular, for ASV sequences obtaining matches with an identity percentage equal or higher than 97% the classification at species level was accepted, otherwise ASVs were classified at higher taxonomic ranks [41]. The ASV table was normalized by using rarefaction for diversity analysis [42]. The Shannon and the Faith Phylogenetic indices [43, 44] were inferred as alpha diversity measure by applying the phyloseq R-package, and statistically relevant differences between groups were evaluated by applying the Wilcoxon test [45]. The principal coordinates analysis (PCoA) that describes the diversity between the samples (i.e. Beta-diversity) based on the weighted and unweighted UNIFRAC metrics, were inferred by using the vegan R package and evaluated by PERMANOVA [46, 47].

The statistical comparison between the healthy and tumor samples was performed by using DESeq2 [48]. To measure differences between tissues in the different conditions, the data were normalized by taking into account inter-sample variability. The p-values obtained were adjusted for multiple comparisons with the Benjamin-Hochberg method. Finally, a supervised model for sample classification was built using the Random Forest (RF) Machine Learning (ML) methods and the R package caret [49]. In particular, the DESeq2 ASVs normalized counts were scaled and centered. Then the dataset was randomly divided into the training set and the test data set that including 54 and 14 samples, respectively. The tuning of RF hyperparameter mtry was performed by repeating cross-validation (10 cross-validation with 10 repeats) on the training dataset and the best mtry value was selected according to ROC metric. Lastly, the accuracy of the RF model was assessed on the test dataset.

## Results

The comparison between the breast tissue microbiota in tumor and that in paired normal adjacent tissues from 34 women affected by breast cancer enabled us to investigate the distribution of microbial communities of each sample. Each sample obtained more than 90% of reads thereby passing quality filtering with an average quality value of 30 (Q30) >80%. The analyzed data were produced by performing an Illumina MiSeq sequencing run, and we obtained a variable number of Paired End (PE) reads per sample (mean 130,820, sd 384,926.925, median 69,920, min 13,417, max 3,215,914). About 96% of input sequences passed the trimming of adaptors and the PCR primer step.

The overall quality of reverse reads was lower than that of forward reads for all the sequenced samples and, in this specific case, did not pass the quality filter in dada2 [50]. To overcome this issue, we applied an approach based on PE reads merging before denoising [51, 52]. About 70% of input reads were successfully merged. The denoising step enabled us to infer the Amplicon Sequence Variants (ASV) sequences and their absolute counts. The ASV sequences were checked to remove chimeras and human contaminants. In order to achieve an adequate compromise between the microbiome sampling and the number of retained samples, the ASV table was rarefied using an equal sequencing depth of 15,000 (Additional File 1: Figure S1), 27 and 16 tumoral and non-tumoral samples were retained, respectively.

**Fig. 1:**
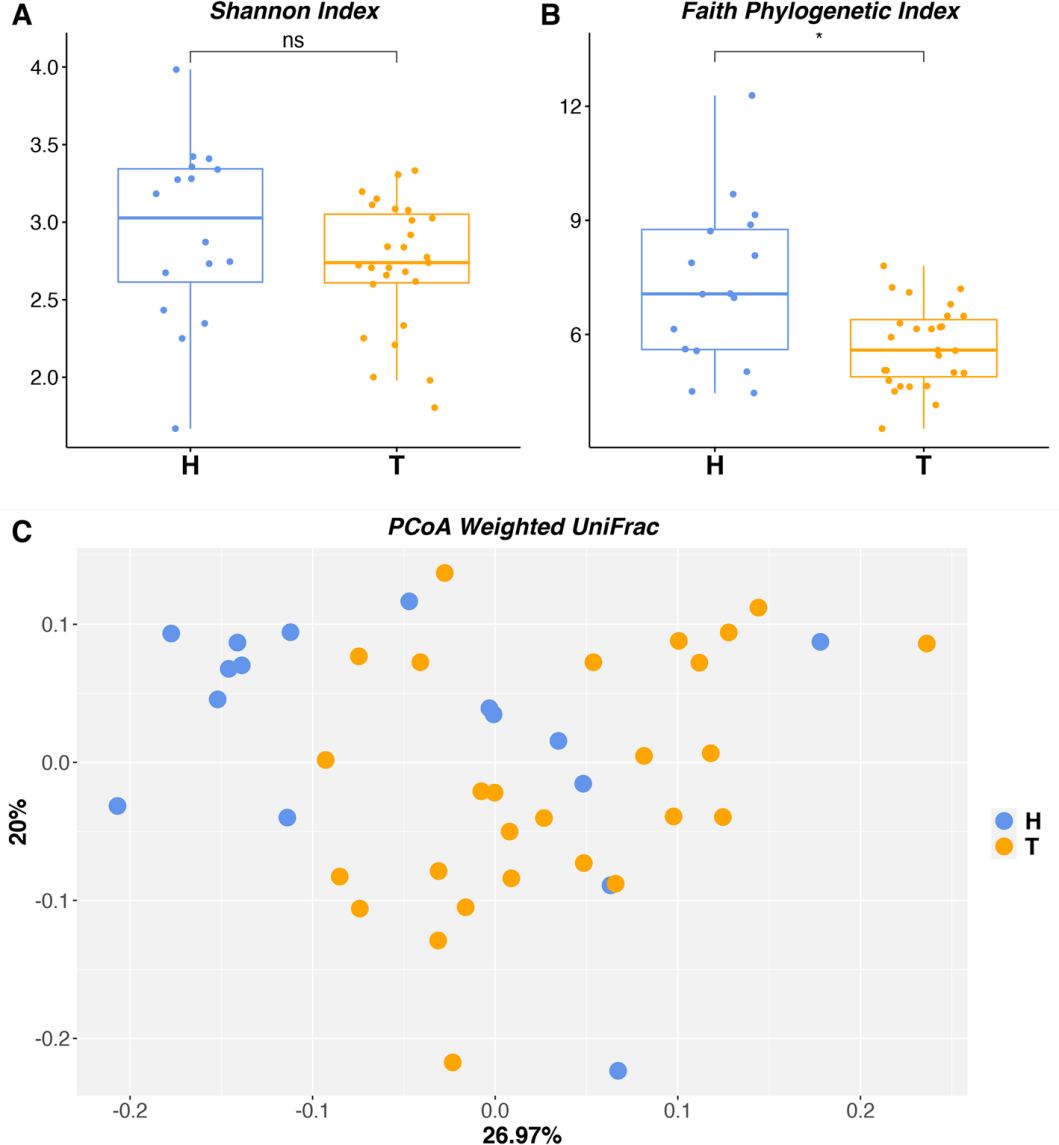
A. The distribution of the inferred Shannon Index for tumoral and non-tumoral samples were shown as boxplot. B. The distribution of the inferred Faith Phylogenetic Index for tumoral and non-tumoral samples were shown as boxplot. C. PCoA plot based on weighted UniFrac measurements. H: healthy tissue; T: tumor tissue

The alpha diversity was measured using the Shannon Index and plotted as a box-plot (Fig. 1a). No statistically significant differences were observed between the tested conditions according to the Shannon index (p-value = 0.1649). Conversely, the distribution of the Faith index differed significantly (p-value ≤ 0.05) between healthy and tumor tissue samples (Fig. 1b).

Although no clear clustering was observed in the PCoA plot based on weighted UniFrac analysis (Fig. 1c) between healthy (H) and tumor (T) tissue samples, the PERMANOVA suggested that about 7% of the observed variability is explained by the conditions (p-value = 0.007). Conversely, neither the PCoA plots nor the PERMANOVA based on unweighted UniFrac (data not reported) resulted in any significant difference between the two conditions (p-value = 0.103).

### Taxonomic Distribution

**Fig. 2:**
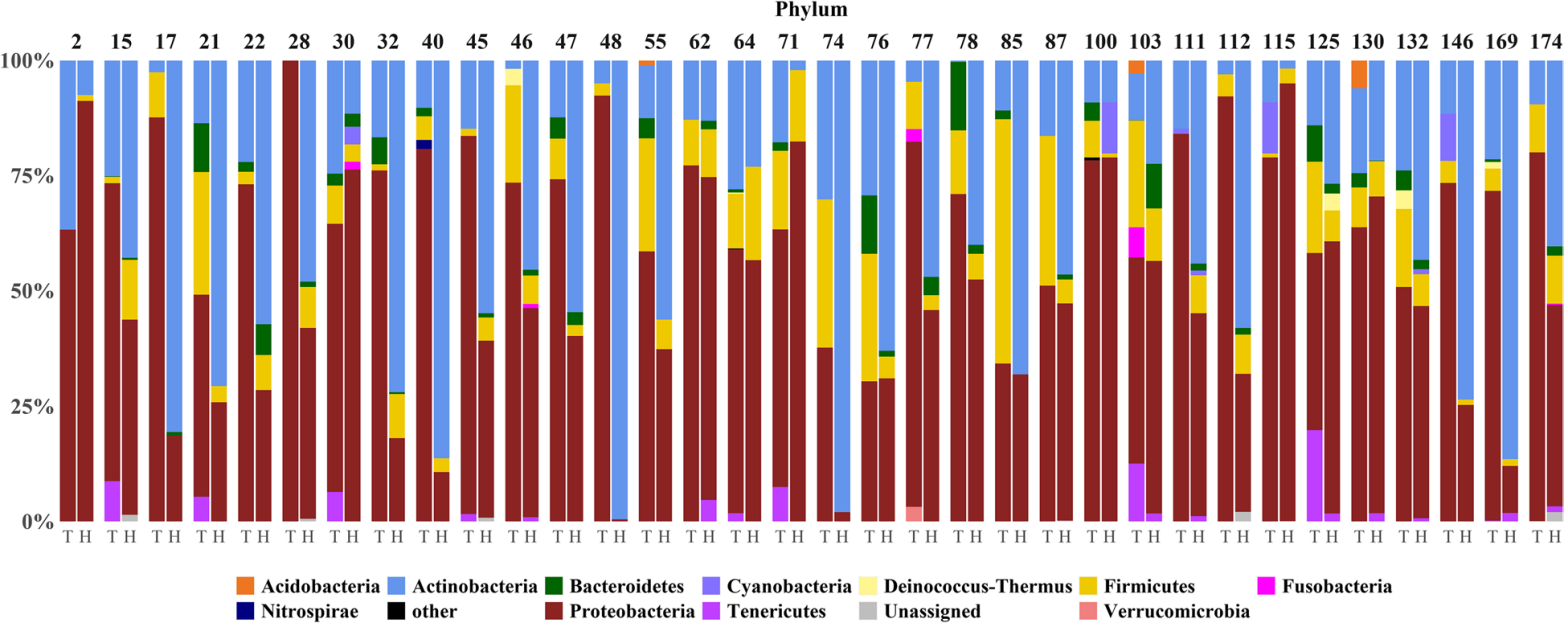
Phyla distribution in healthy (H) and tumor (T) samples are show per each enrolled subject as stacked bar-plot. All the rare taxa are collapsed in “other” (relative abundance < 1% in all samples)

All the ASVs were taxonomically annotated at least at kingdom level. Generally, 13 phyla, 25 classes, 59 orders, 105 families, 199 genera and 514 species were identified across all samples. The distribution of phyla is shown in Fig. 2. The most predominant phyla are Actinobacteria and Proteobacteria (about 31% and 55.4% on average, respectively). Gammaproteobacteria (40.22%), Actinobacteria (25.09%), Bacilli (7.83%) and Alphaproteobacteria (5.57%) are the most abundant classes among all tumor and normal samples.

**Fig. 3:**
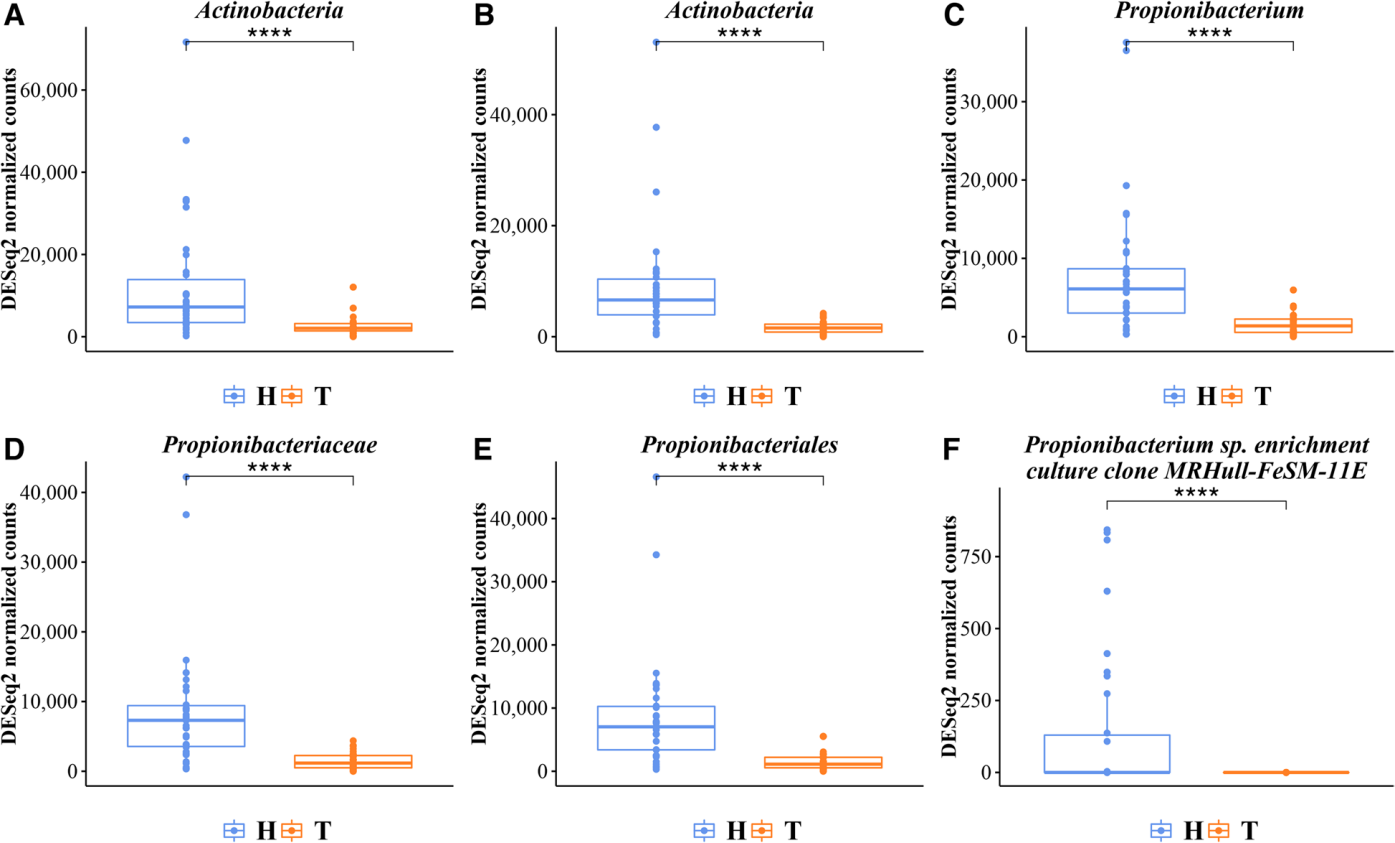
(panels** A-F**). Normalized read counts distribution of statistically different taxa between healthy and tumor samples were shown as box-plot. In detail, Actinobacteria at phylum level, p-value=1,36E-11; Actinobacteria at class level, p-value=3,23E-12; *Propionibacterium* at genus level, p-value=3,39E-06; Propionibacteriaceae at family level, p-value=7,24E-08; Propionibacteriales at order level, p-value=2,06E-11; *Propionibacterium_sp._enrichment_culture_clone_MRHull-FeSM-11*, at species level, p-value=2,28E-35. (Other box plots are shown in Additional File 1, Figure S1)

**Fig. 4:**
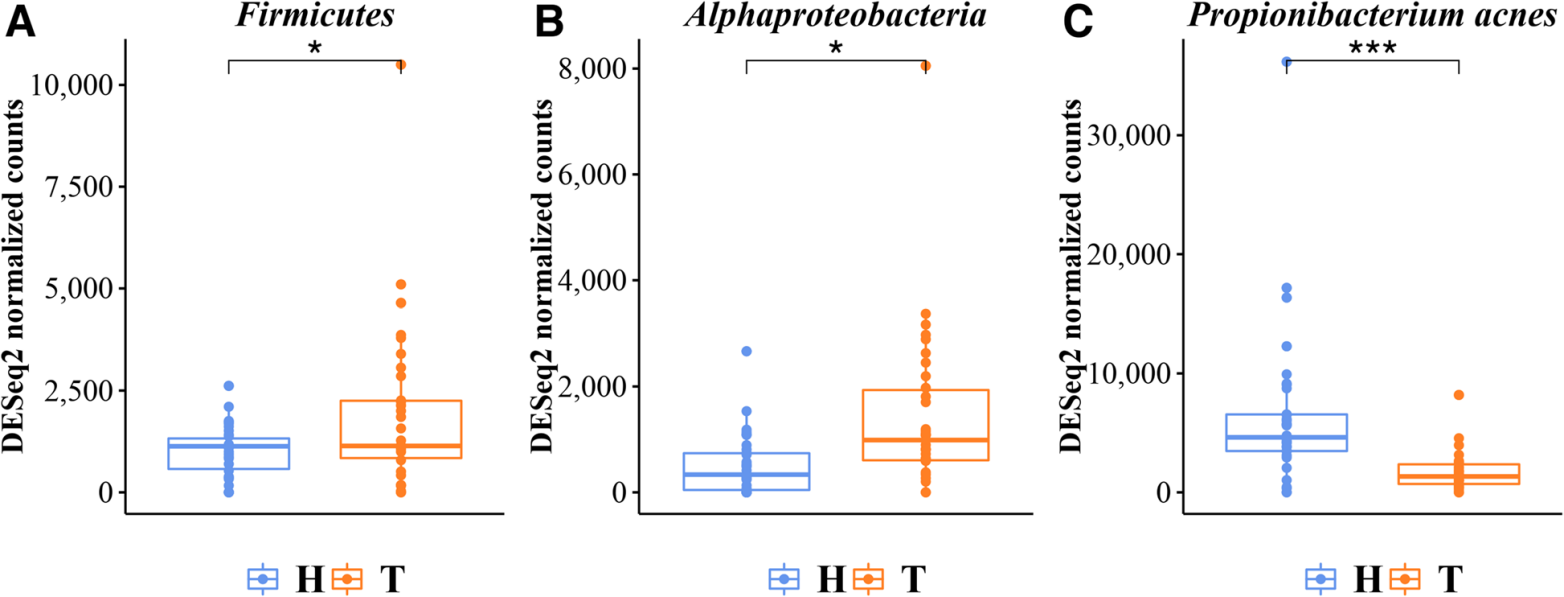
(Panels **A**-**C**). Normalized read counts distribution of statistically different taxa between healthy and tumor samples were shown as box-plot. Firmicutes at phylum level, p-value=0,047230491; Alphaproteobacteria at class level, p-value=0.014872974; *Propionibacterium_acnes* at species level, p-value=0,0005835

The most prevalent families are Propionibacteriaceae (23.57%), Moraxellaceae (17.83%) and Pseudomonadaceae (15.19%). The genera *Propionibacterium* (22.59%), *Acinetobacter* (15.43%) and *Pseudomonas* (15.10%) are the most abundant. The results of statistical comparisons are reported in Table 3. The box-plot of each statistically different taxon between healthy and tumor samples, are shown in Fig. 3 (A-F) and Fig. 4 (A-C), and in Additional File 1: Figure S2. Overall, in non-tumoral paired samples a higher abundance of taxa belonging the Actinobacteria phylum was found. In particular, the order Propionibacteriales, the family Propionibacteriaceae, the genus *Propionibacterium* and species *Propionibacterium sp. enrichment culture clone MRHull-FeSM-11R* and *Propionibacterium acnes* are more abundant in non-tumoral tissues (Fig. 3 A-F and Fig. 4 C). Conversely, Firmicutes and Alpha-proteobacteria are significantly overrepresented in tumoral tissues.

**Table 3:** List of taxa that differ significantly between healthy and tumor samples

Taxa	*Significance*	*Log2 fold change*	*Adjusted p-value*
***Phylum***			
* Actinobacteria*	****	2.28	1.36e^-11^
* Firmicutes*	*	-0.89	0.047
***Class***			
* Actinobacteria (Class)*	****	2.51	3.23e-^12^
* Alphaproteobacteria*	*	-1.51	0.015
***Order***			
* Propionibacteriales*	****	2.53	2.06e-^11^
* Aeromonadales*	****	26.23	1.99e^-19^
* Selenomonadales*	****	25.6	2.17e^-18^
***Family***			
* Propionibacteriaceae*	****	2.54	7.24e^-08^
* Aeromonadaceae*	****	25.18	4.60e^-21^
***Genus***			
* Propionibacterium*	****	2.36	3.39e^-06^
* Aeromonas*	****	26.32	1.00e^-19^
***Species***			
* Variovorax sp. WO3*	****	25.13	1.28e^-18^
* Moraxella sp. S2*	****	-25.4	1.42e^-17^
* Pseudomonas sp. PS9 (2007)*	****	25.14	4.39e^-21^
* Propionibacterium sp.* *Enrichment culture clone MRHull-FeSM-11E*	****	27.3	2.28e^-35^
* Pseudomonas sp. IMER-A2-21*	****	26.39	9.32e^-22^
* Pseudomonas brenneri*	****	-25.87	4.13e^-18^
* Neisseria elongata*	****	25.00	4.22e^-17^
* Propionibacterium acnes*	***	1.91	0.006

The analysis was performed by comparing healthy and tumor samples, consequently if the log2 fold change is positive, the taxon counts are higher in healthy than in tumor samples. Significance level refers to adjusted p-values: * ≤ 0.05, ** ≤ 0.01, *** ≤ 0.001, **** ≤ 0.0001

**Fig. 5:**
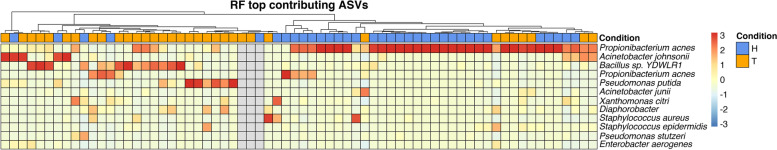
Heatmap showing top 12 important ASVs that contribute most to the RF Classification Model. The species listed represent the deepest taxonomic classification rank of each ASV. Samples are shown in column and clustered by using the Ward’s method for hierarchical clustering relying on Euclidean distances [53]

In order to identify the ASVs able to discern among tumoral and non-tumoral tissues by using a robust and reliable method, a supervised classification machine learning model was built using Random Forest (RF). To avoid overfitting and to properly train the model, the dataset was divided into a training and a test dataset, accounting for 54 and 14 samples, respectively. The overall accuracy of the test dataset was about 89%, with two misclassifications for healthy samples. The ASVs that most contribute to the model accuracy were selected and used to plot a heatmap (Fig. 5).

As shown in Fig. 5, two main clusters may be identified, the first one is constituted mainly by healthy tissues and the second one by tumor tissues. The first ASV was assigned to *Propionibacterium acnes* and was principally observed in healthy tissues. This result agrees with those obtained by comparing taxa abundances in DESeq2.

Regarding BRCA mutational status, there were only three BRCA-positive patients in our population and in particular one carrying a mutation in the *BRCA1* gene and two in *BRCA2* gene. Consequently, the data were not enough to carry a reliable statistical analysis. Similarly, the same issue was observed for other confounding factors, i.e. smoking status and contraception usage.

## Discussion

Studies of the entire microbial communities and their relationships with the host have been conducted to evaluate how their imbalance could be involved in health maintaining and diseases [20, 54–61]. In particular, several studies have linked the microbiome to the initiation and progression of different types of cancer, including breast cancer [58, 59]. Moreover, the cooperation of microbial communities’ imbalance with diet, obesity, estrogens and immune modulation has been considered an important promoter of breast cancer [12, 62]. Notably, the majority of authors [16–19, 24, 36, 37, 61–64] note that their findings are hypothesis-generators and support further investigations to identify a microbial risk signature for breast cancer and potential microbial-based prevention and/or therapies.

In this scenario, we studied the resident breast microbiota in tumor and paired normal breast tissue from 34 BC patients. The aim of our study was to evaluate the microbial composition of breast tumor tissues and healthy tissues in the attempt to shed light on the link between dysbiosis and breast cancer which, in turn, may indicate that a change in bacterial species could contribute to the modulation of cancer development. A comparison between paired healthy and tumor tissues revealed differences of bacterial community and composition. The number of ASVs detected between paired normal and tumor tissue showed significant differences in richness between the sampled communities. Proteobacteria and Actinobacteria showed differences between two groups: healthy tissues showed an increase of Actinobacteria and a decrease of Proteobacteria; the opposite appeared in tumor tissues. Conversely, in healthy tissues, appear to be more prevalent *Propionibacterium* and *Pseudomonas*.

In particular, we observed an overall decrease of microbial alpha diversity in tumoral tissues compared to healthy ones. We also found a significant depletion of *Propionibacterium acnes* in tumor tissues versus normal breast tissues, which is a novel finding. *Propionibacterium acnes* (currently denominated *Cutibacterium acnes*) is a component of the human microbiome found in several body districts. Its over-representation in normal tissue was observed by comparing abundances (DESeq2) and also by machine learning (Random Forest), which indicates that these results are robust. This gram-positive species is considered an opportunist pathogen because potentially pathogenic genes were found in the genomes available (5 phylotypes). However, the role of *Propionibacterium* remains to be established. For example, Talib et al. 2015 [65] described a potential antitumoral action of *Propionibacterium acnes* in breast cancer, and Portillo et al. 2013 [66] suggested that it plays a role in implant-associated infections.

Our study supports the presence of microbial DNA in breast tissues that could probably influence the local tissue microenvironment. In the attempt to minimize any external variations (including sample preparation and sequencing) between healthy and tumor tissues, we compared healthy tissues to the paired tumor breast tissues taken from each woman at the same time and in the same conditions. All 68 samples were amplified, purified and sequenced together in a single sequencing run in order to minimize any analytical variation. Although, our results are at variance from those reported by others [16–19, 24, 61, 62], it is important to highlight that differences in both experimental procedures (i.e., primer design and the use of bioinformatic pipelines to filter and to analyze data) and different cohort enrolled can affect results and, therefore, their comparison. Survey results about the breast cancer tissue microbiome, are reported for a more comprehensive comparison (Additional File 1: Table S1) and it is important to note how several factors, such as ethnicity, dietary habits, geographical origin, lactation status, pharmaco-therapeutic before surgery, the method of sample collection [66, 67] can affect the composition of microbial tissues [16]. For instance, *Fusobacterium nucleatum* has been described as a key player in several pathological conditions, and particularly in colon rectal cancer. However, earlier work was principally based on a comparison between healthy and unhealthy samples [16–19, 24], not including paired tissues analysis.

Accordingly, another key difference is that the primer pairs we used differed from those used in other studies. In their review of the association between the gut/breast microbiota and breast cancer, Laborda-Illanes et al. 2020 [20] highlighted the differences among studies in terms of data results. We counted 6 different combinations of the 16 S hypervariable region in 10 papers (i.e., V4=3, V6=2, V3-V4=1, V3-V5=2, V1-V2=1 and V3=1). Consequently, it may be misleading to compare surveys conducted using different marker regions, also considering the different efficiency in target amplification and in the resolution of taxonomic assignment.

Therefore, it is difficult and also controversial to define a precise signature of the breast cancer microbiome. Thus, our effort was not to define a universal bacterial signature in breast cancer tissues, but to reinforce the concept that it is an altered balance that characterizes tumor tissues versus healthy tissues in the same woman, also at the very close proximity regions, which *per se* increases significance of the microbial presence at the level of breast tissue cell transformation. Indeed, we also found that microbial alpha diversity was overall lower in tumor tissues than in healthy tissues.

Larger studies, conducted in diverse geographic regions, are needed to define - if existing - a precise bacterial signature for each type of tissue neoplasia and thus to determine the role played by the microbiome in breast cancer onset and development. Furthermore, it is difficult to use general approaches in different cohorts particularly those living in different geographic regions. Rather, it may be more effective to study patients, cohort-by-cohort or groups of subjects living in the same region and under similar environmental conditions. It is also necessary to understand, using in vitro systems as human tumoroids and mouse models, how different pre-surgery antibiotic regimens can induce disturbances in the breast microbiota and how these disturbances affect BC progression. Indeed, the lack of this information may represent a limitation.

It is now necessary to understand the effect that the metabolites produced from resident bacteria have on the development and progression of the breast. However, it is necessary not only to study the association among microbiota, tumor development and progression and/or anti-tumor immune responses using metagenomic sequencing technologies, but also to demonstrate microbiota functionality using transcriptional and/or metabolic profiling [68, 69], thereby paving the way to the application of further precision medicine in BC patients.

## Conclusions

This study reveals a highly significant difference in the abundance of the various taxa of the microbiome in breast tumor tissues versus their healthy tumor-adjacent counterparts in women after surgery. These alterations reflect qualitative and quantitative differences of taxa, thus indicating their relevance in the comprehension of microbiome content and their role in tumor tissues.

Finally, assessing the different microbial composition in relation to BC onset and progression could be a goal to achieve in future studies on more numerous cohorts of patients.

## Supplementary Information

**Additional file 1:** **Figure S1.** Rarefaction curves used to define the rarefaction threshold. **Figure S1.** shows box-plot of statistically different taxa between healthy and tumor samples. **Table S1.** lists the differences among data obtained in different cohorts of patients in several studies by different Authors for results comparison.

## Data Availability

The raw sequencing data are available in the Sequence Read Archive (SRA) repository under the BioProject PRJNA759366.

## Abbreviations

ASVs: Amplicon Sequence Variants
BC: breast cancer
ER: estrogen receptor
ML: Machine Learning
NGS: next generation sequencing
PE: paired end
RF: Random Forest
