# Supplementary material for: Microbiome composition indicate dysbiosis and lower richness in tumor breast tissues compared to healthy adjacent paired tissue, within the same women
Source: BMC Cancer. 2022 Jan 3;22:30. doi: 10.1186/s12885-021-09074-y (PMC8722097; doi:10.1186/s12885-021-09074-y)
Supplement: Supplementary file 1 — Additional file 1: Figure S1. Rarefaction curves used to define the rarefaction threshold. Figure S1. shows box-plot of statistically different taxa between healthy and tumor samples. Table S1. lists the differences among data obtained in different cohorts of patients in several studies by different Authors for results comparison. [file 12885_2021_9074_MOESM1_ESM.docx]

**Supplementary Informations**

Microbiome composition indicate dysbiosis and lower richness in tumor breast tissues compared to healthy adjacent paired tissue, within the same women

Maria Valeria Esposito^1,2†^, Bruno Fosso^3†^, Marcella Nunziato^1,2†^, Giorgio Casaburi^4^, Valeria D'Argenio^1,2,5^, Alessandra Calabrese^1,2,6^, Massimiliano D'Aiuto^6,7^, Gerardo Botti^8^, Graziano Pesole^3,9,^* and Francesco Salvatore^1,2,^*

^1^Department of Molecular Medicine and Medical Biotechnologies, University Federico II, Via Sergio Pansini, 5, 80131, Napoli (NA), Italy

^2^CEINGE - Biotecnologie Avanzate, Via Gaetano Salvatore, 486, 80145, Napoli, (NA); Italy

^3^Institute of Biomembranes, Bioenergetics and Molecular Biotechnologies, Consiglio Nazionale delle Ricerche; Via Giovanni Amendola, 122/O, 70126, Bari (BA), Italy

^4^Evolve Biosystems, Inc, Davis, CA 95618 USA

^5^Department of Human Sciences and Quality of Life Promotion, San Raffaele Open University, Via di Val Cannuta, 247, 00166, Rome, (RM), Italy

^6^Department of Senology, Istituto Nazionale Tumori - IRCCS, 'Fondazione Pascale', Via Mariano Semmola, 53, 80131, Napoli (NA), Italy

^7^Clinica Villa Fiorita, Via Filippo Saporito, 24, 81031, Aversa (CE), Italy

^8^Scientific Directorate, Istituto Nazionale Tumori, Fondazione G. Pascale, IRCCS, Via Mariano Semmola, 53, 80131, Napoli (NA), Italy

^9^Department of Biosciences, Biotechnology and Biopharmaceutics, University of Bari "A. Moro", Piazza Umberto I, 1, 70121, Bari (BA), Italy

† Co-first authors

*Co-corresponding authors: salvator@unina.it; g.pesole@ibiom.cnr.it

| **Content** |  |
| --- | --- |
| **Supplementary Figure S1** | **Pag. 2** |
| **Supplementary Figure S2** | **Pag. 3** |
| **Supplementary Table S1** | **Pag. 4** |
| **References** | **Pag. 4** |

**
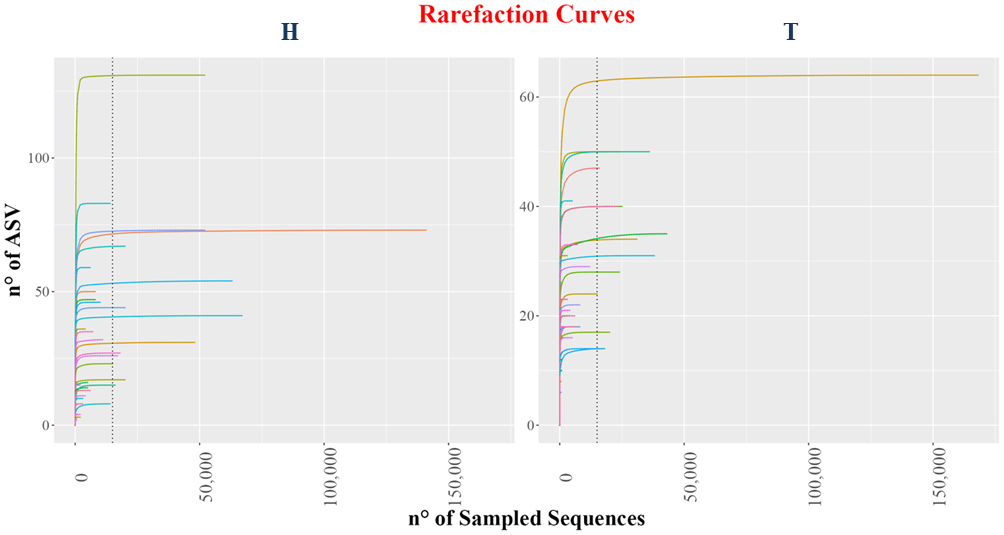
**

**Supplemental Figure S1**. Rarefaction curves representing the number of ASVs observed at an increasing number of sampled sequences. A dotted black line at 15.000 sampled sequences to show the used rarefaction threshold.


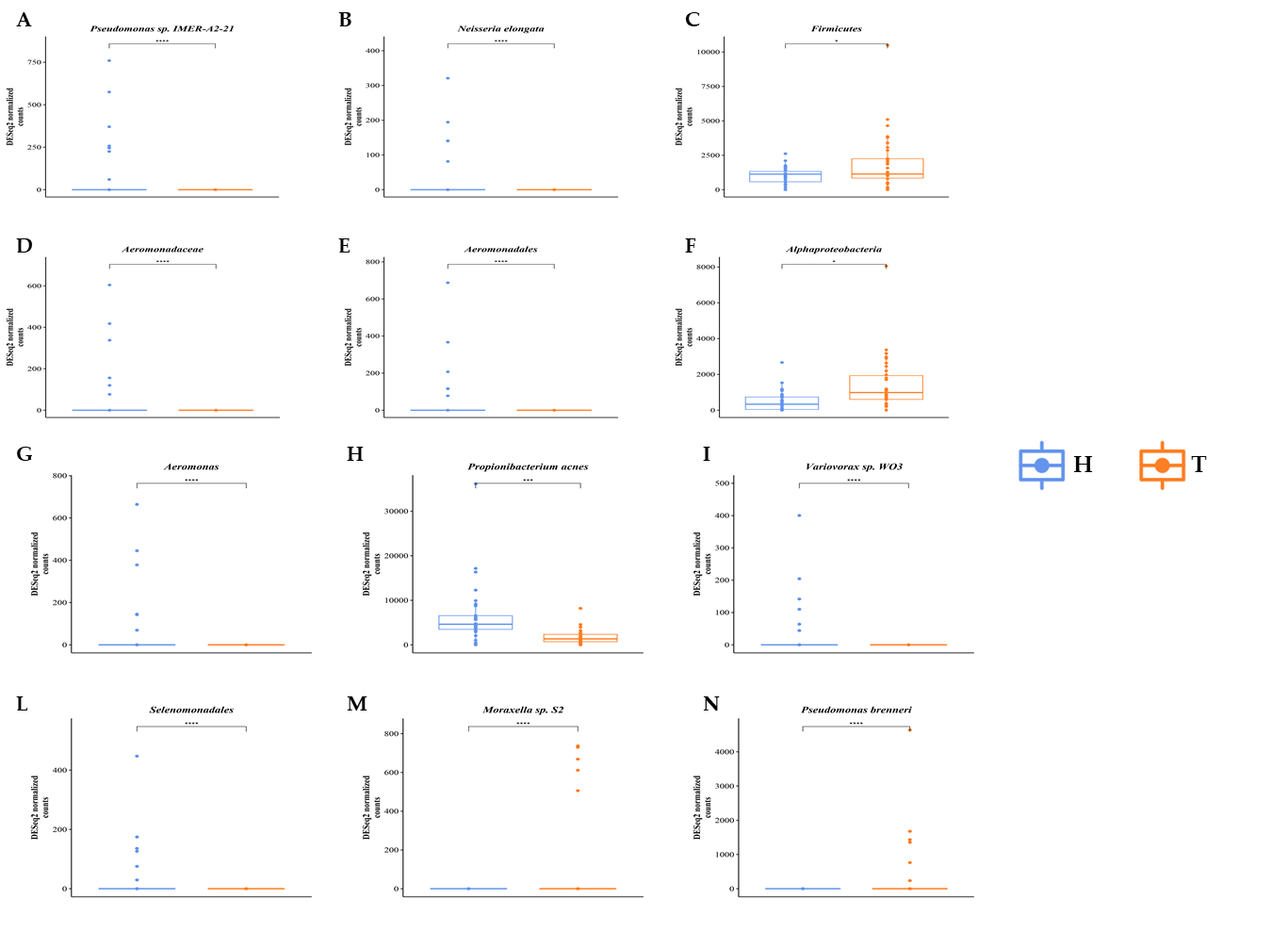


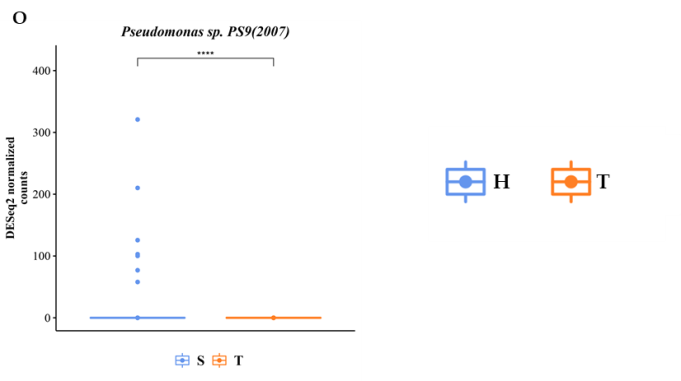


**Supplemental Figure S2**. (panels A-O). Box-plot of statistically different taxa between healthy (in blue) and tumor samples (in orange). In particular: Pseudomonas_sp._IMER-A2-21, at species level, p-value=9,32E-22; Neisseria_elongata, species, p-value=4,22E-17; Aeromonadaceae, at family level, p-value=4,60E-21; Aeromonadales, at order level, p-value=1,99E-19; Aeromonas, at genus level, p-value=1,00E-19; Variovorax_sp._WO3at species level, p-value=1,28E-18; Selenomonadales, at order level, p-value=2,17E-18; Moraxella_sp._S2, at species level, p-value=1,42E-17; Pseudomonas_brenneri, at species level, p-value=4,13E-18; Pseudomonas_sp._PS9(2007), at species level, p-value=4,39E-21.

**Supplemental Table S1.** Comparison among studies of the microbiome of breast cancer tissues.

| **References** | **Xuan, et al., 2014 [36]** | **Urbaniak, et al., 2014 [18]** | **Hieken, et al., 2016 [17]** | **Wang, et al., 2017 [19]** | **Meng, et al., 2018 [24]** | **Costantini L. et al., 2018 [37]** | **Smith, et al. 2019 [16]** |
| --- | --- | --- | --- | --- | --- | --- | --- |
| **Features** |  |  |  |  |  |  |  |
| **Number of analyzed patients** | n=20 | n=81 | n=28 | n=78 | n=94 | n=16 | n=72 |
| **Paired Tissue*** | **Yes (n=20)** | No | No | No | No | **Yes (n=16)** | **Yes (n=11)** |
| **Not paired tissue (healthy women mostly cosmetic surgery)** | None | n=36: benign pathologies, and cosmetic surgery | n=13 | n=21 | n=22 | None | n=8 |
| **Sources of the study cohorts** | USA (CA) | Canada and Ireland | USA (MN, FL) | USA (OH) | China | Italy (Rome Area) | USA (TN) |
| **Methodology** | 16S V4 rDNA (Illumina) | 16S V6 rRNA (Ion Torrent) | 16S V3-V5 rDNA (Illumina) | 16S V3-V4 rRNA (Illumina) | 16S V1-V2 rRNA (Illumina) | 16S V3 rRNA (Ion Torrent) | 16S V4 rRNA (Illumina) |
| **Pipeline for data analysis** | QIIME | UCLUST | IM-TORNADO | UCLUST | QIIME | ION reporter software | QIIME |
| **Novelty** | Quantitative PCR to determine bacterial copy number | Immunofluorescence and DNA damage assay | Other samples (skin and buccal swab) | Other samples (urine, oral swab) | Metabolic KEGG analysis | Other samples (Core Needle and Surgical Excision Biopsies) | Racial differences |
| **Results (microbial composition)** | Increase of *Proteobacteria, Firmicutes, Actinobacteria, Bacteroidetes and Verrucomicrobia* in BC. Increased *Sphingomonas yanoikuyae* in normal tissues and *Methylobacterium radiotolerans* in tumor. | Increase of *Proteobacteria,* *Firmicutes* and *Escherichia coli* in all BC samples. Increase of *Bacillus* and *Acinetobacter* in BC in Canadian women. Increase of *Enterobacteriaceae* and *Staphylococcus* in women with BC in IrelandBC. | Increase of *Fusobacterium, Atopobium, Gluconacetobacter, Hydrogenophagaand Lactobacillus* in BC tissues. | Increase of: *Corynebacterium, Staphylococcus, Actinomyces* and *Propionibacteriaceae* in BC; decrease of: *Methylobacterium* | Increase of: *Propionicimonas, Micrococcaceae, Caulobacteraceae, Rhodobacteraceae, Nocardioidaceae, Methylobacteriacea* and *Agrococcus*in BC; decrease of: *Bacteroidaceae* | Increase of *Ralstonia* in BC. No significant differences between healthy adjacent breast tissues and BC tissues. | Increase of: *Pseudomonadaceae* in normal pair and tumor tissues. Increase of: *Ruminococcaceae,* *Fusobacteria* and *Spirochetes* in BC. Increase of: *Pseudomonadaceae, Sphingomonadaceae,* *Caulobacteraceae;* decrease of: *Ruminococcaceae* and *Clostridia* in normal pairs; Increase of: *Actinomycetaceae* and decrease of: *Pseudomonadaceae, Sphingomonadaceae* and *Ruminococcaceae* in normal tissues. |

***Tumor and normal adjacent breast tissues taken from the same woman and checked by histopathological analysis.**

**References**

16. Smith A, Pierre JF, Makowski L, Tolley E, Lyn-Cook B, Lu L, et al. Distinct microbial communities that differ by race, stage, or breast-tumor subtype in breast tissues of non-Hispanic Black and non-Hispanic White women. Sci Rep. 2019;9:1–10.

17. Hieken TJ, Chen J, Hoskin TL, Walther-Antonio M, Johnson S, Ramaker S, et al. The microbiome of aseptically collected human breast tissue in benign and malignant disease. Sci Rep. 2016; 6:30751.

18. Urbaniak C, Gloor GB, Brackstone M, Scott L, Tangney M, Reida G. The microbiota of breast tissue and its association with breast cancer. Appl Environ Microbiol. 2016;82:5039–48.

19. Wang H, Altemus J, Niazi F, Green H, Calhoun BC, Sturgis C, et al. Breast tissue, oral and urinary microbiomes in breast cancer. Oncotarget. 2017;8:88122–38.

24. Meng S, Chen B, Yang J, Wang J, Zhu D, Meng Q, et al. Study of microbiomes in aseptically collected samples of human breast tissue using needle biopsy and the potential role of in situ tissue microbiomes for promoting malignancy. Front Oncol. 2018;8:318.

36. Xuan C, Shamonki JM, Chung A, DiNome ML, Chung M, Sieling PA, et al. Microbial dysbiosis is associated with human breast cancer. PLoS One. 2014;9:e83744.

37. Costantini L, Magno S, Albanese D, Donati C, Molinari R, Filippone A, et al. Characterization of human breast tissue microbiota from core needle biopsies through the analysis of multi hypervariable 16S-rRNA gene regions. Sci Rep. 2018;8:4–12.
